# Differences in the Treatment Response to Antithyroid Drugs versus Electroconvulsive Therapy in a Case of Recurrent Catatonia due to Graves' Disease

**DOI:** 10.1155/2012/868490

**Published:** 2012-06-12

**Authors:** Takahiro Saito, Rie Saito, Hiroshi Suwa, Fumiatsu Yakushiji, Kenji Takezawa, Mitsuru Nakamura

**Affiliations:** ^1^Department of Psychiatry, Yokohama Camellia Hospital, 920 Shiranecyo, Asahiku, Kanagawa, Yokohama 241-0003, Japan; ^2^Department of Psychiatry, Oishi Kinen Hospital, 2-23-1 Nishiaraihoncyo, Adachi, Tokyo 123-0845, Japan; ^3^Department of Psychiatry, Tokyo Metropolitan Health and Medical Treatment Corporation Ebara Hospital, 4-5-10 Higashiyukigaya, Ota, Tokyo 145-0065, Japan; ^4^Department of Internal Medicine, Tokyo Metropolitan Bokuto Hospital, 4-23-15 Koutoubashi, Sumida, Tokyo 130-8575, Japan; ^5^Department of Psychiatry, Matsuzaki Hospital, 67 Sanbongicyomotosanbongi, Aichi, Toyohashi 441-8152, Japan; ^6^Department of Psychiatry, Tokyo Metropolitan Health and Medical Treatment Corporation Toshima Hospital, 33-1 Sakaecyo, Itabashi, Tokyo 173-0015, Japan

## Abstract

We reported a case which presented recurrent episodes of catatonia as a result of Graves' disease with hyperthyroidism. The patient showed different treatment response in each episodes; in the first episode, psychiatric and physical symptoms were resolved by a combination of antithyroid and anxiolytic therapies, while in the second episode, the combination therapy did not ameliorate her symptoms and ECT was indicated. We postulated that decreased CSF level of TTR and the resulting susceptibility to the derangement of peripheral thyroid function might be involved in this different treatment response.

## 1. Introduction

Since von Basedow described a psychotic illness in a patient with an exophthalmic goiter [1], an association between hyperthyroidism and psychiatric syndromes has been under clinical attention. A combination of the treatment of underlying thyroid illnesses and antipsychotic therapy is the first-line treatment option of psychiatric symptoms in most cases. Previous reports suggest, however, in several cases merely a partial response might be obtained from the combination; and further intensive treatments are required. We therefore present a case of recurrent catatonia due to Graves' disease with hyperthyroidism which responded to antithyroid therapy in the first episode but required electroconvulsive therapy (ECT) after a relapse. We then review literatures and discuss the possible mechanism which caused a different treatment response between the two episodes.

## 2. Case Presentation

Mrs. A, a 25-year-old woman, was first referred to a B psychiatric clinic in April 1996 presenting thought disturbance, irritability, impulsive behavior, and excitement. She had good premorbid function and had no history of drug abuse. A year earlier, after the delivery of her first daughter, she had complained of finger tremors but had not sought medical care. She was diagnosed with atypical psychosis and was put on a course of antipsychotic therapy, without response. Meanwhile, she developed a fever and tachycardia and was admitted to a C general hospital for scrutiny of her symptoms. 

On admission, she manifested stupor, catalepsy, echolalia, and intermittent excitement. A physical examination found sinus tachycardia, goiter, and proptosis. Laboratory studies revealed elevated free triiodothyronine (FT3) 12.9 pg/ml (reference range: 2.50~5.50 pg/mL), elevated free thyroxine (FT4) 46.53 ng/dL (reference range: 0.85~1.80 ng/dL), and suppressed thyroid stimulating hormone (TSH) level (<0.03 *μ*IU/mL. reference range: 0.50~4.00 *μ*IU/mL). Together with elevated titers of TSH antibodies 20.6% (reference range: <15%), these findings were consistent with Graves' disease with hyperthyroidism. Given the patient's clinical presentation and the result of laboratory findings, we rendered a diagnosis of catatonia due to Graves' disease with hyperthyroidism. Thiamazole (TMZ) 60 mg/d, diazepam 10 mg/d for catatonic stupor, and metoprolol 60 mg/d for tachycardia were prescribed. Over a period of a month, her psychiatric and physical manifestations had gradually subsided. However, a follow-up laboratory study indicated a subnormal range of FT3 and FT4; thus the dose of TMZ was progressively decreased. She recovered euthyroid and was discharged on the 65th hospital day. 

For the following 10 years, Mrs. A regularly visited her family physician receiving a continuation therapy of TMZ 5 mg every other day, without any recurrence of symptoms. However, in October 2006, at age 35, she complained of tachycardia and insomnia. Though these symptoms had barely interfered with her everyday functioning for a period of 3 months, on the 1st of February 2006, she abruptly uttered incoherent speech. She was referred to a D psychiatric clinic and was started on antipsychotic therapy, in vain. On the 13th of February, her psychiatric symptoms were aggravated and she was admitted to a psychiatric ward of an E general hospital. 

During inpatient evaluation, she presented an unintelligible soliloquy and also exhibited excitement, stereotypy, and mannerism. Physical examination revealed no characteristic findings of hyperthyroidism other than sinus tachycardia. Slight elevation of FT4 (1.56 ng/dL) was evident in laboratory studies. We considered the possible relapses of catatonia due to Graves' disease and added potassium iodide 100 mg/d for the elevated FT4 levels and diazepam 20 mg/d for catatonic symptoms in the regimen. On the 8th hospital day, despite the normalization of FT4 levels, she still manifested an alteration of catatonic excitement and stupor. Treatment with diazepam was discontinued and replaced with haloperidol 20 mg/d. Although she showed slight improvement in response, antipsychotic therapy did not elicit remarkable relief to her catatonic symptoms. Being considered refractory to drug therapy, electroconvulsive therapy (ECT) was recommended and consent was obtained from her husband.

She was started on a course of bitemporal ECT and given pulse wave stimuli using ThymatronR (Somatics Inc., Lake Bluff, IL). After the 3rd trial, she showed remarkable improvement in catatonic symptoms and spontaneously complained of auditory hallucination. We continued ECT with a combination therapy of olanzapine 20 mg/d, and a full resolution of psychiatric symptoms was achieved after the 7th trial. Meanwhile, we increased TMZ to 20 mg/d and added levothyroxine sodium 25 *μ*g/d for the maintenance of euthyroid status. After being discharged on the 49th hospital day, she regularly visited the outpatient clinic of a C general hospital, where her antipsychotics had been tapered and discontinued. She now receives antithyroid drugs and remains euthyroid without any recurrence of psychiatric symptoms.

## 3. Discussion

The present paper illustrated a recurrence of catatonic episodes associated with hyperthyroidism due to Graves' disease, which responded to antithyroid therapy in the first episode but required ECT when relapsed. Previous reports indicated that a spectrum of major psychiatric illnesses, including depression, mania, and schizophreniform psychosis, were related to hyperthyroidism [2]. Though in most cases a combination of neuroleptic treatment and antithyroid therapy had benefits to relieving their psychiatric manifestations, at least three case reports described patients with acute psychosis due to new-onset Graves' hyperthyroidism who had only a partial response to antithyroid therapy and required a course of ECT [3]. However, to our knowledge, there are few reports which documented the difference in treatment response of the same patient suffering recurrent episodes of a similar psychiatric picture with underlying aggravation of thyroid function, after an interval of 10 years.

In both episodes, the clinical presentation of our case fulfilled diagnostic criteria of International Statistical Classification of Diseases and Related Health Problems Tenth Revision (ICD-10) for organic catatonic disorder (F06.1). However, we observed a difference in the degree of thyroid dysfunction and physical symptoms between the two circumstances. In the first episode, an elevation of both FT3 and FT4 in association with a suppression of TSH was found and the patient exhibited cardinal features of hyperthyroidism such as tremor, tachycardia, goiter, and proptosis. By contrast, only a mild elevation of FT4 and tachycardia as a physical sign were observed in the second episode. We postulate that this difference might have resulted in the altered responsiveness to the antithyroid therapy in our case. 

One possible mechanism is the derangement of thyroid hormone homeostasis in the central nervous system (CNS). Thyroid hormone function in the CNS is in part regulated by thyroid hormone transporters in the blood-brain barrier (BBB) and blood-cerebrospinal fluid (CSF) barrier. Of note, reduced CSF levels of transthyretin (TTR), a major carrier of T4 in the BBB and blood-CSF barrier, has been implicated in depression and first-onset psychosis, respectively [4, 5]. Joo et al. revealed in laboratory mouse experiments that chronic immobilization stress induced depression-like behaviors and a reduced level of TTR in the mouse's cortex [6]. It is speculated that the first catatonic episode of our case might have exacerbated CSF level of TTR and thus affected the intractable CNS homeostasis of thyroid hormone even after the recovery of the peripheral euthyroidism in the second episode. It is also postulated that disturbance in thyroid hormone homeostasis in the CNS might modulate the responsiveness of catecholamine receptors, as stated in a hypothesis of thyroid-catecholamine-receptor interaction [7]. Further studies with large number of participants are worthwhile to investigate whether aforementioned reduction of CSF TTR level would be observed in the group of psychotic patients with Graves' disease.
